# Supplementary material for: The Assessment of Activity of Antiseptic Agents against Biofilm of Staphylococcus aureus Measured with the Use of Processed Microscopic Images
Source: Int J Mol Sci. 2022 Nov 4;23(21):13524. doi: 10.3390/ijms232113524 (PMC9658380; doi:10.3390/ijms232113524)

Table S1. Confluence [%] of analyzed staphylococcal biofilms (number of strains = 10)

strain description	confluence [%]	average confluence [%] for n=10 strains
ATCC 6538	99,9	93,61±5,55
ATCC 33591	99,9	
S1	81,7	
S2	95,7	
S3	94,4	
S4	97,8	
S5	95,3	
S6	91,2	
S7	90,8	
S8	89,4	

Table S2. The Fluorescence Intensity of live and dead (L, D, respectively) staphylococcal biofilms with regard to their location in **T** (top), **M** (middle) and **B** (bottom) parts of biofilm

**Strain no**	**Part of biofilm**	**Fluorescence Intensity L:D**	**Matches pattern FI M > FI T and FI B?**
**ATCC 6538**	T	980: 710	**Yes**
	M	2499:1441	
	B	258: 742	
**ATCC 33591**	T	492: 938	**Yes**
	M	891: 689	
	B	161:591	
**S1**	T	664:523	**Yes**
	M	1725:1705	
	B	326:623	
**S2**	T	1353:779	**Yes**
	M	4130:3174	
	B	703:1021	
**S3**	T	954:627	**Yes**
	M	4265:3094	
	B	498:295	
**S4**	T	769:412	**Yes**
	M	1900:621	
	B	219:492	
**S5**	T	205:474	**Yes**
	M	432:381	
	B	176:321	
**S6**	T	522:634	**Yes**
	M	1421:839	
	B	283:425	

**S7**	T	405:174	**Yes**
	M	571:331	
	B	108:273	
**S8**	T	411:290	**Yes**
	M	710:629	
	B	138:305	

Figure S1. Three types (A,B,C) of distribution of dead/cell wall compromised cells vs. viable/cell wall non-compromised cells in staphylococcal biofilm *in vitro*; the vertical cross-section through the biofilm structure. A – strain ATCC 6538; B – strain S1, C -ATCC 33591. Scale bar is 30 µm. Microscope SP8, magn.40x.

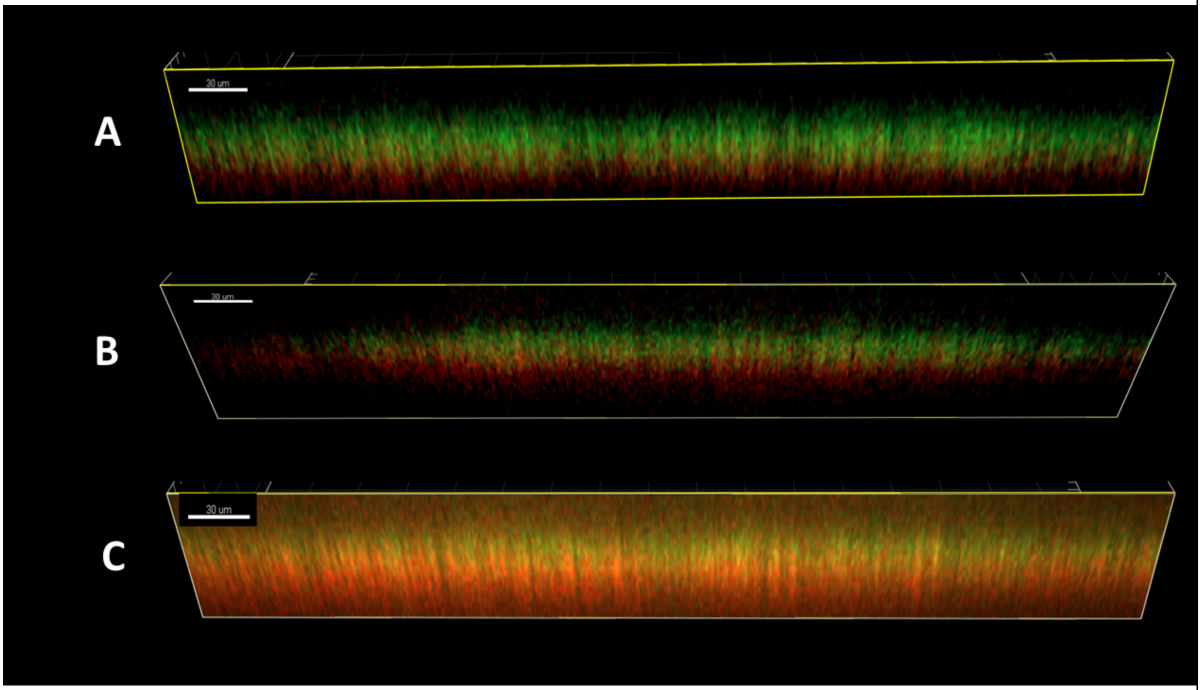

Figure S2. The different types of Live/Dead cells' distribution in biofilm within single plate. Strain ATCC 33591. Scale bar is 40μm

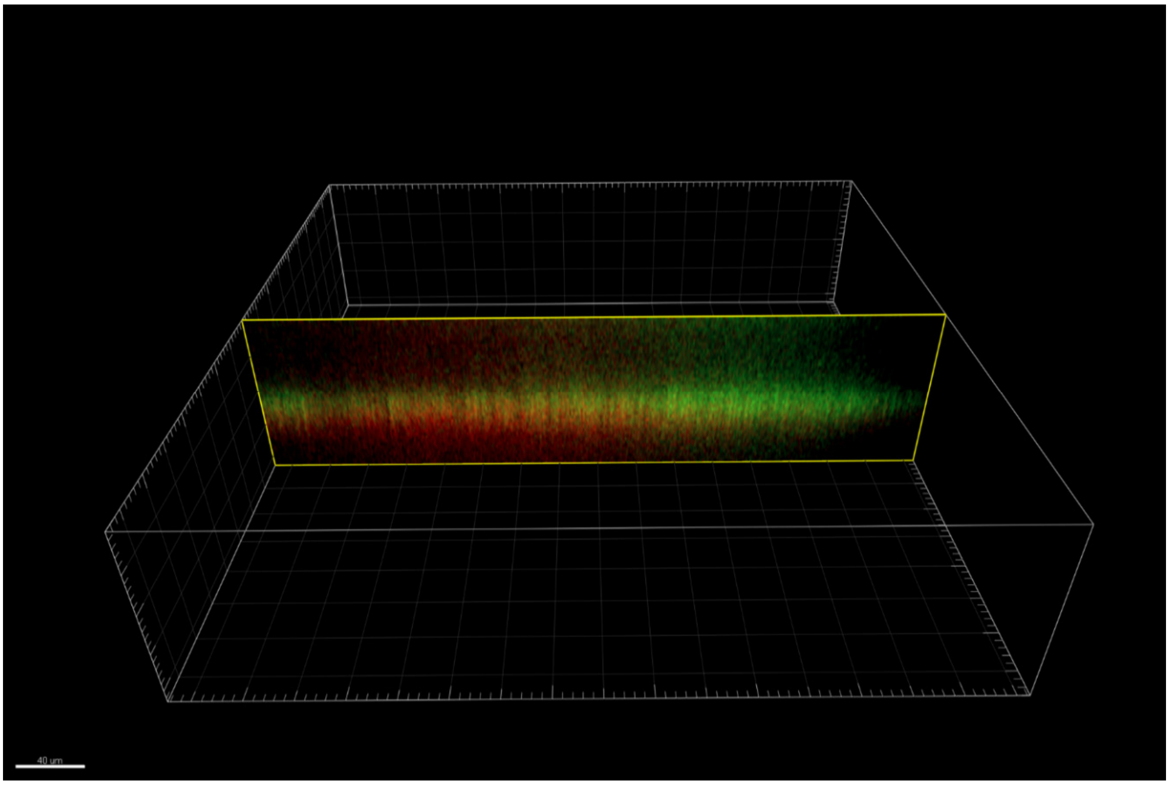

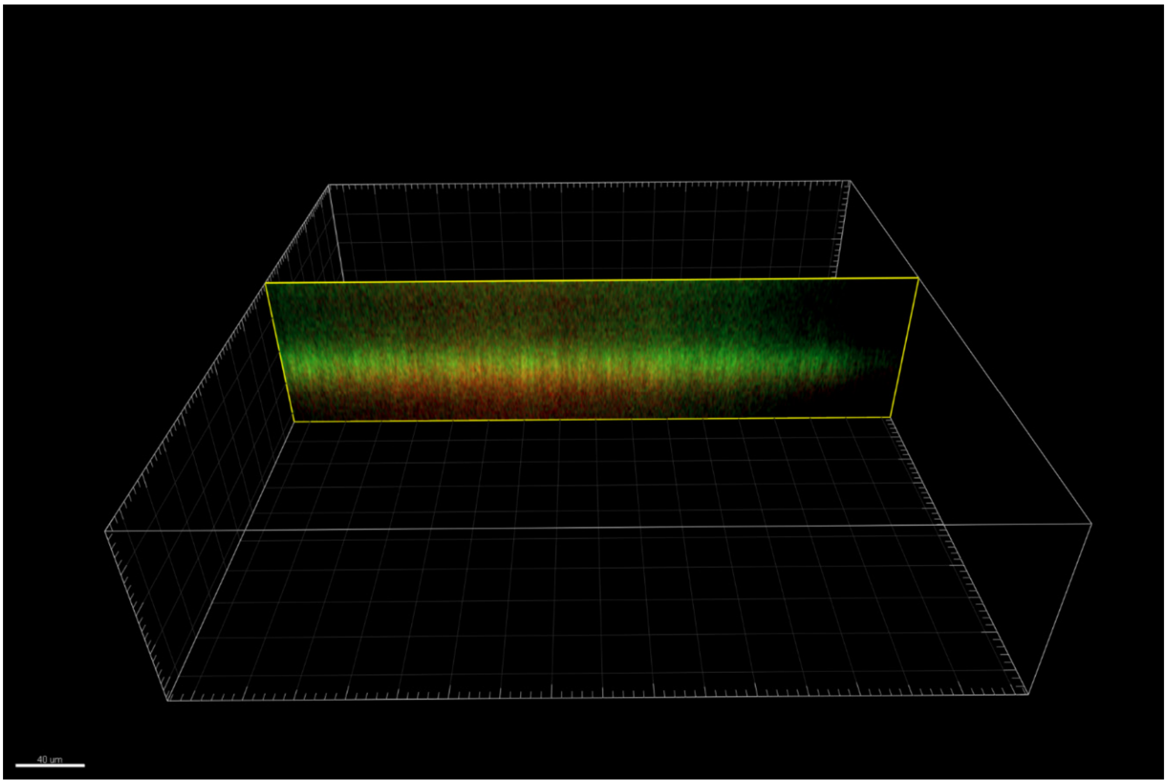

Figure S3. The impact of operators' various pipetting habits (A,B,C) on dye distribution in the well (of a 24-well plate) covered with biofilm formed by the same staphylococcal strain (ATCC 6538). Picture D presents the biofilm of the aforementioned strain dyed with the L/D method by an operator with 3 years of experience in staphylococcal biofilm culturing and dyeing. The red cross indicates the approximate place of pipette tip placement.

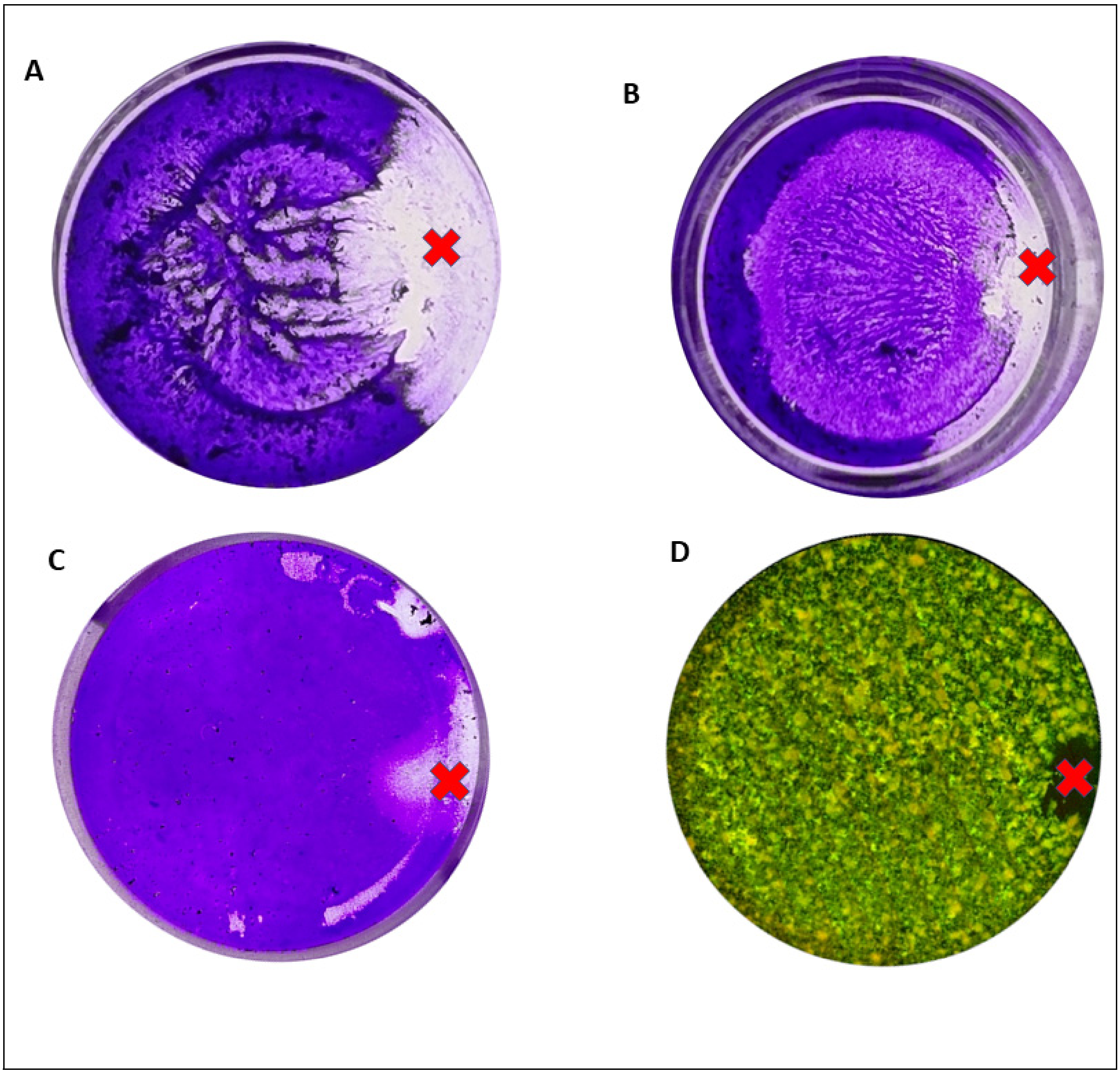

Supplement: Supplementary file 1 [file ijms-23-13524-s001.zip › ijms-2013835-supplementary.pdf]
